# Surface of CuO Nanoparticles Modified by *p*-Benzoquinone for N_2_-Selective Membrane

**DOI:** 10.3390/membranes12121229

**Published:** 2022-12-05

**Authors:** Juyeong Lee, Hiesang Sohn, Sang Wook Kang

**Affiliations:** 1 Department of Chemical Engineering and Materials Science, Sangmyung University, Seoul 03016, Republic of Korea; 2 Department of Chemical Engineering, Kwangwoon University, Seoul 01897, Republic of Korea; 3 Department of Chemistry and Energy Engineering, Sangmyung University, Seoul 03016, Republic of Korea

**Keywords:** polyvinylpyrrolidone, selectivity, permeance, composite membrane, interaction, separation performance

## Abstract

In this study, CuO nanoparticles and *p*-benzoquinone (*p*-BQ) were added to a polyvinylpyrrolidone (PVP) matrix to increase N_2_/CO_2_ selectivity. The added *p*-BQ allowed CuO to be distributed in a uniform size in the PVP/CuO composite membrane and the matrix to be flexible by forming the interaction with PVP. The surface modification of CuO by *p*-BQ and the well-dispersed size affected the increase in the separation performance. The PVP/CuO/*p*-BQ composite membranes showed an N_2_/CO_2_ selectivity of about 23.1 with N_2_ permeance of about 13.3 GPU, while the separation performance of PVP was not observed. The enhanced separation performance is attributable to the surface of CuO nanoparticles modified by *p*-BQ inducing CO_2_ molecules to be relatively slowly transported by the adsorption properties in the polymer matrix. The chemical properties and coordinative interaction for PVP/CuO/*p*-BQ composite membrane were measured by FT-IR spectroscopy, thermogravimetric analysis, UV–vis, scanning electron microscopy, X-ray photoelectron spectroscopy, and transmission electron microscopy.

## 1. Introduction

The increase in carbon dioxide emissions is closely related to the history of human economic development. Carbon dioxide emissions have increased with industrial developments. Among these developments, the size of the economy, industrial structure, and carbon emission intensity have a close influence on carbon dioxide emission. The best method to reduce carbon emissions is industrial restructuring. The current focus is on optimization to reduce carbon emissions in industrial structures [1]. If the carbon dioxide in the atmosphere generated by industrial structures is not reduced, it will have a significant impact on marine life. The increase in carbon dioxide changes the balance of carbonate chemicals and decreases the pH of the ocean surface, which affects the initial development and lifespan of marine animals and changes the marine ecosystem significantly as the concentration of carbon dioxide in atmosphere increases [2,3]. This increased concentration of carbon dioxide affects climate change as well as marine ecosystems. Currently, the greenhouse effect caused by carbon dioxide is increasing, and global warming is considered as a major problem among climate problems. Global warming is the result of carbon dioxide and methane in the atmosphere. In other words, an increase in carbon dioxide emission will lead to an increase in global warming [4], which is expected to cause significant problems in the global climate [5]. Filters are used not only to separate substances that cause climate change, but also to filter substances that cause air pollution. These days, as interest in COVID-19 increases worldwide, concern about masks and ventilators is also increasing. There is an urgent need for research on a technology for capturing inhalable particulate matter present in the air. In order to protect the human respiratory system from ultrafine dust and viruses such as COVID-19, research on masks for various purposes is being actively conducted. Filter membranes are commonly used to improve the filter efficiency of masks and have excellent separation performance when separating harmful substances. Currently, research on reusable masks and filters of various materials is being conducted, as well as research improving the separation performance of harmful substances using membrane-based technology [6]. Furthermore, on-body analysis using body fluids can provide various health-related information of users, and wearable sensors using such fluids are being developed. In the process, research on the material development of the converter used in manufacturing wearable sensors is also underway [7]. On the other hand, the most notable technology for reducing CO_2_ generated in industrial structures is the application of membrane technology for greenhouse gas separation/capture [8,9]. There are various technologies available, such as absorption using a solvent or solid absorbent, pressure and temperature adsorption using various solid adsorbents, cryogenic distillation, and membranes [10,11]. Technologies using membranes are generally used for CO_2_ separation by manufacturing membranes based on a polymer. The components constituting the membrane are dissolved in a polymer on one side and transported through the membrane as a result of the concentration gradient. The principle of membrane gas separation uses a method of separating gas by selectively transporting gas components from one side of the membrane to the other [12,13]. Unlike in the separation process, the main role of the membrane in gas absorption is that it provides physical support and a large interface area between the gas and the liquid [14]. Problems to be researched relating to these membranes include plasticization resistance, chemical resistance, long-term stability, and cost. As for the application of membranes, there is a gas absorption method and a separation method for the membranes. As a method of separating CO_2_ from the mixed gas, there is also a method of manufacturing a membrane using an ionic liquid such as a supporting film and a poly (ionic liquid) film. The method for manufacturing a membrane using ionic liquid has more advantages than the method using a conventional, volatile organic solvent. This is because it has combustibility, higher thermal stability, ease of recycling, and can lower energy demand for solvent regeneration. However, there are also obvious drawbacks to the method for the manufacture of a membrane using ionic liquid. The high viscosity, high production cost, and unclear toxicity of the ionic liquid can be disadvantages [15,16]. Unlike this method, there is also a method of preparing a membrane as a multilayer, composite membrane. The multilayer composite is a film that can independently optimize different functions to reduce overall transport resistance and has few restrictions on material mechanical properties and processability. However, it has disadvantages regarding the problems of composite membranes that are commonly discussed, such as plasticization and aging [17]. On the other hand, mixed-matrix membrane (MMM) technology has also been studied to develop a cost-effective and high-performance separator for CO_2_ by synthesizing it through partial acid hydrolysis based on polyvinylamine (PVAm). MMM technology is attracting attention as a technology to exceed the trade-off limit [18]. Furthermore, a membrane for increasing CO_2_/N_2_ permeability and selectivity using multi-walled carbon nanotubes (MWCNTs) and cellulose acetate (CA) has been reported. In this experiment, it was found that, in the CO_2_/N_2_ separation experiment, the separation performance of MMMs may vary with the amount of CA and may improve by up to 40 GPUs of selectivity [19]. Based on the polyimide membrane, nanocrystalline UiO-66 and its derivatives were developed through a functionalization step before synthesis and then integrated into a polymer membrane to develop an MMM. Thereby, the CO_2_ separation performance was improved. This experiment sought to increase the separation performance of CO_2_/N_2_ by adding nanocrystalline UiO-66; however, the separation performance of CO_2_/N_2_ increased, but the CO_2_/N_2_ selectivity decreased. It was found through experiments that, when the UiO-66 derivative with different functional groups was used, the solubility of N_2_ was suppressed by other functional groups, and, thus, the CO_2_/N_2_ permeance was increased by about 12% compared to the previous one [20]. A method of improving CO_2_/N_2_ separation using an ionic liquid was also used. Research was also conducted to evaluate the effect of poly (ionic liquids) (PILs)-based copolymer on the preparation and the performance of membrane for CO_2_/N_2_ separation. In this study, it can be seen that, when a homogeneous, free-standing solid membrane was manufactured with a combination of different weights between poly (vinylimidazolium)–polystyrene copolymers, the permeance of CO_2_ reached 16.5~24.5 and CO_2_/Nity 31.7~34.4 [21]. Research using a nitrile-containing anion paired with 1-alkyl-3-methylimidazolium cations has shown that nitrile groups can be introduced to optimize CO_2_/N_2_ separation. In this experiment, we found that the permeability of 1-ethyl-3-methylimidazolium tetracyanoborate improved by 30% compared to the popular penetration rate [22]. Various methods such as using ionic liquid and manufacturing MMMs are being studied to manufacture the membrane to improve CO_2_/N_2_ separation performance. 

Recently, based on the PEO polymer, 4-hydroxybenzoic acid was added to increase CO_2_ separation performance. As a result, the CO_2_ separation performance increased from 1.8 to 23. It can be seen that the separation performance increased due to the role of the barrier to the gas transport of benzene rings in 4-hydroxybenzocyanate [23]. Furthermore, an ionic liquid of 1-butyl-3-methylimidazolium tetrafluoroborate was added to the CO_2_ transport membrane to generate CuO nanoparticles in the ionic liquid. The CuO nanoparticles increased the CO_2_/N_2_ separation performance by generating the interaction between the ionic liquid and the CuO. In this experiment, it can be seen that CO_2_ permeance was 52.4 PGU, CO_2_/N_2_ selectivity was 21.0, and, in the case of the neat BMIM^+^BF_4_^−^ membrane, CO_2_ permeance was 17.0 GPU and CO_2_/N_2_ selectivity was 5.0 [24]. On the other hand, a composite membrane was prepared using 1-butyl-3-methylimidazolium tetrafluoroborate with ZnO nanoparticles. It can be seen that the permeance of CO_2_ and the CO_2_/N_2_ separation performance was increased by 1-butyl-3-methylimidazolium tetrafluoroborate to induce an increase in CO_2_ solubility. In the case of the composite membrane including ZnO nanoparticles, it can be seen that CO_2_/N_2_ selectivity and CO_2_ permeance were improved from 5.0 and 17.0 GPUs to 42.1 and 101 GPUs, respectively, compared to the neat BMIM^+^BF_4_^−^ membrane [25]. On the other hand, since it is known that *p*-BQ can polarize metal surfaces such as Ag or Cu, it can be utilized for modification of particles. Thus, surface CuO nanoparticles modified by *p*-benzoquinone (*p*-BQ) were utilized for highly N_2_-selective membranes since CO_2_ molecules can be affected by adsorption properties in the polymer matrix. 

## 2. Materials and Methods

### 2.1. Materials

Polyvinylpyrrolidone (PVP) (Mw = 360,000) and 1,4-benzoquinone (98%) were purchased from JUNSEI (Tokyo, Japan). Copper(Ⅱ) oxide (CuO) was purchased from Aldrich Chemical Co (St. Louis, MO, USA). Ethanol (94.5%) was purchased from Daejung Chemicals & Metals Co., Ltd (Seoul, Korea) and commercial macroporous polysulfone was purchased from Toray Advanced Materials Korea Inc (Seoul, Korea).

### 2.2. Methods

#### 2.2.1. Membrane Preparation

PVP/CuO/1,4-benzoquinone composite membranes were prepared as follows. PVP was dissolved into ethanol in an amount of 3 wt% solutions based on the solvent. The membrane ratio of PVP/CuO/1,4-benzoquinone was varied at a ratio of 1:2.5:0.25. CuO was dissolved in ethanol by an amount according to the corresponding ratio, sonicated for 4 min with a Sonifier 450 (Branson), and TCNQ and PVP were added into the solution. The mixture was then stirred for one hour. The prepared solution was coated on a macroporous polysulfone support using an RK control coater (Model K202, Control RK Print-Coat Instruments LTD (Litlington, UK). This coated membrane was dried in a constant-temperature and constant-humidity chamber for 3 min.

#### 2.2.2. Material Characterization

The SEM image was measured using a JEOL JSM-5600LV (Akishima, Tokyo, Japan). IR spectra were measured using a VERTEX 70-FT IR spectrometer (Billerica, MA, USA); 16 scans were signal-averaged with a resolution of 4 cm^−1^. The thermal stability of the membrane with increasing temperature was analyzed using thermogravimetric analysis (TGA Q50M, TA instruments, New Castle, DE, USA). UV was measured using a Genesys 10S UV–vis spectrometer (ThermoFischer, Waltham, MA, USA).

#### 2.2.3. Gas Separation Experiments

The prepared membrane was measured immediately after drying in a constant-temperature and constant-humidity chamber. The gas permeation experiment was conducted by permeating a single gas into the membranes, respectively. The pressure of each gas was 2 bar, and the permeance was measured using a bubble flowmeter. A gas permeation unit (GPU) is 1 × 10^−6^ cm^3^ (STP)/(cm^2^ s cm Hg).

## 3. Results and Discussion

### 3.1. Separation Test

The permeances of CO_2_ and N_2_ were measured to confirm the effect of CuO and *p*-BQ on separation performance as shown in Table 1. It is known that a neat PVP membrane shows no separation performance since CO_2_ and N_2_ have similar permeance. The change in separation performance caused by adding CuO and *p*-BQ to neat PVP was measured. When CuO was added to neat PVP, the permeance of both CO_2_ and N_2_ increased, and, thus, the separation performance was not observed [26]. However, when both CuO and *p*-BQ were applied, the separation performance increased to about 23.1. The permeance to N_2_ was not changed, as it was before when *p*-BQ was added, but the separation performance increased since the permeance to CO_2_ decreased. 

### 3.2. Scanning Electron Microscopy

**Figure 1:**
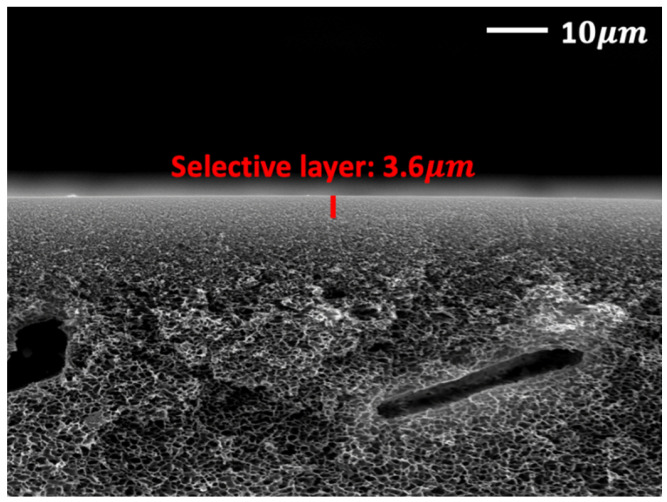
SEM image: PVP/CuO/*p*-BQ composite membrane coated onto polysulfone support.

Figure 1 shows the thickness of the PVP/CuO/*p*-BQ composite film coated on the porous polysulfone support. The measured thickness of the PVP/CuO/*p*-BQ film was about 3.6 μm. 

### 3.3. FT-IR Spectroscopy

**Figure 2:**
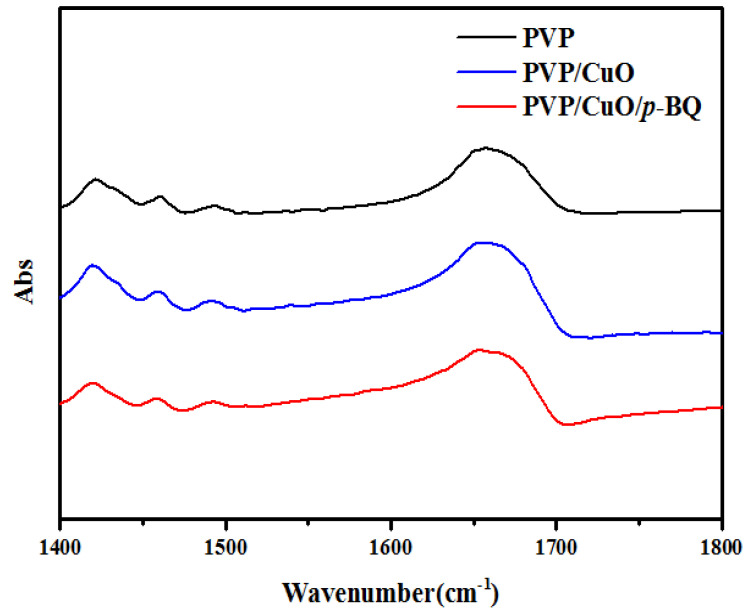
IR spectra of PVP, PVP/CuO, and PVP/CuO/*p*-BQ composites.

**Figure 3:**
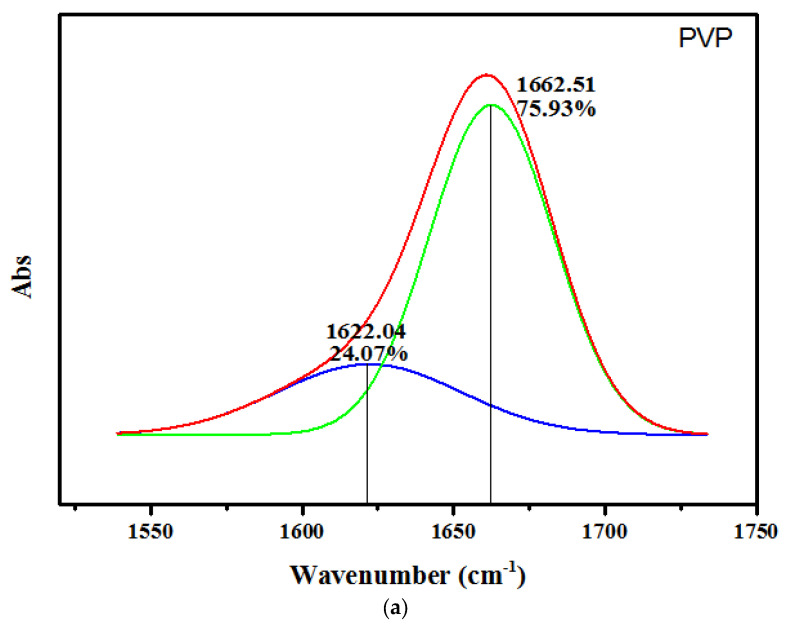

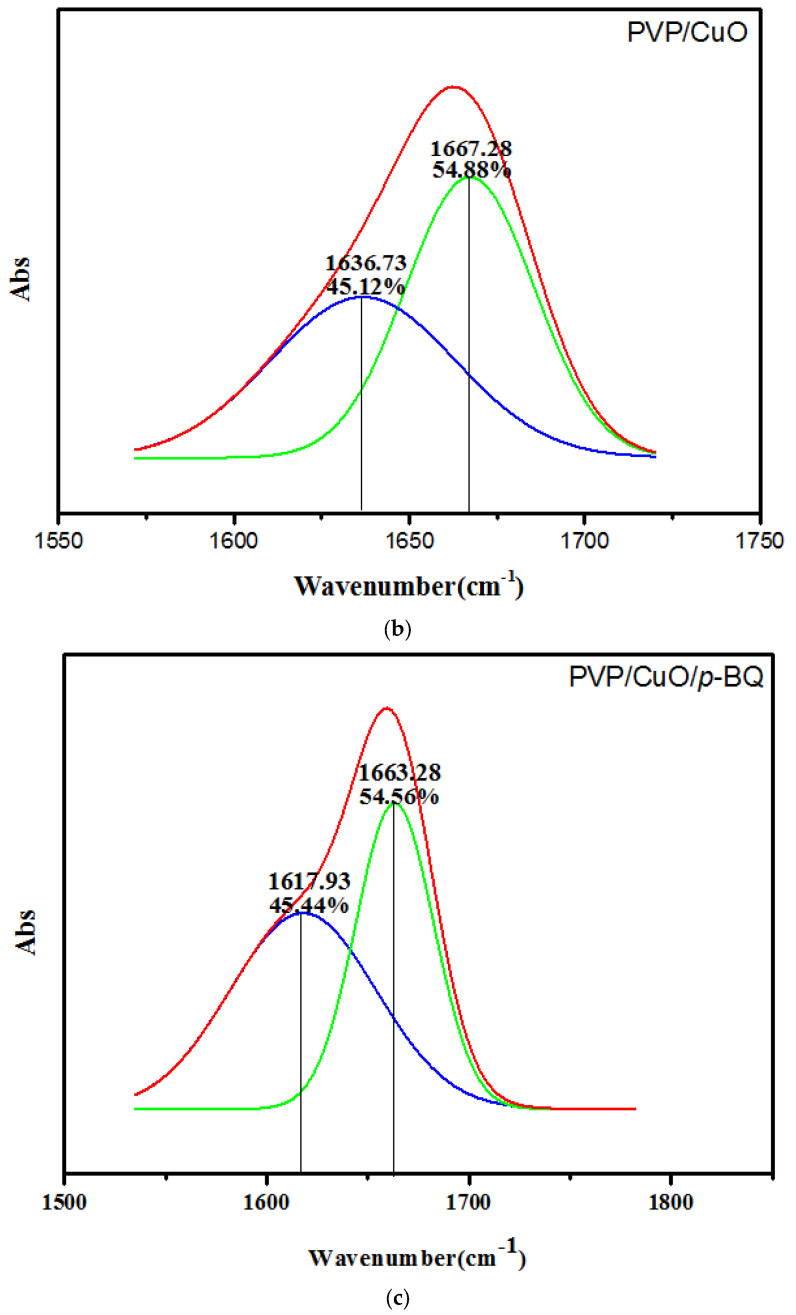
(**a**) Deconvoluted IR spectra of PVP, (**b**) deconvoluted IR spectra of PVP/CuO composite, (**c**) deconvoluted IR spectra of PVP/CuO/*p*-BQ composite.

The interaction between CuO and *p*-BQ in PVP/CuO and PVP/CuO/*p*-BQ was measured using FT-IR by adding CuO and *p*-BQ to neat PVP. Figure 2 and Figure 3 show an absorption band at about 1660 cm^−1^ for the case of neat PVP. When CuO was applied, the wavenumber was higher than that of neat PVP, as shown in Figure 2 and Figure 3. In the case of PVP/CuO/*p*-BQ, it was observed that the wavenumber was lower than that of PVP/CuO and exhibited a slightly larger absorption band than the wavenumber of neat PVP. The incorporation of CuO changed the interaction between the chains, but it seems that it was not large.

### 3.4. Thermogravimetric Analysis

**Figure 4:**
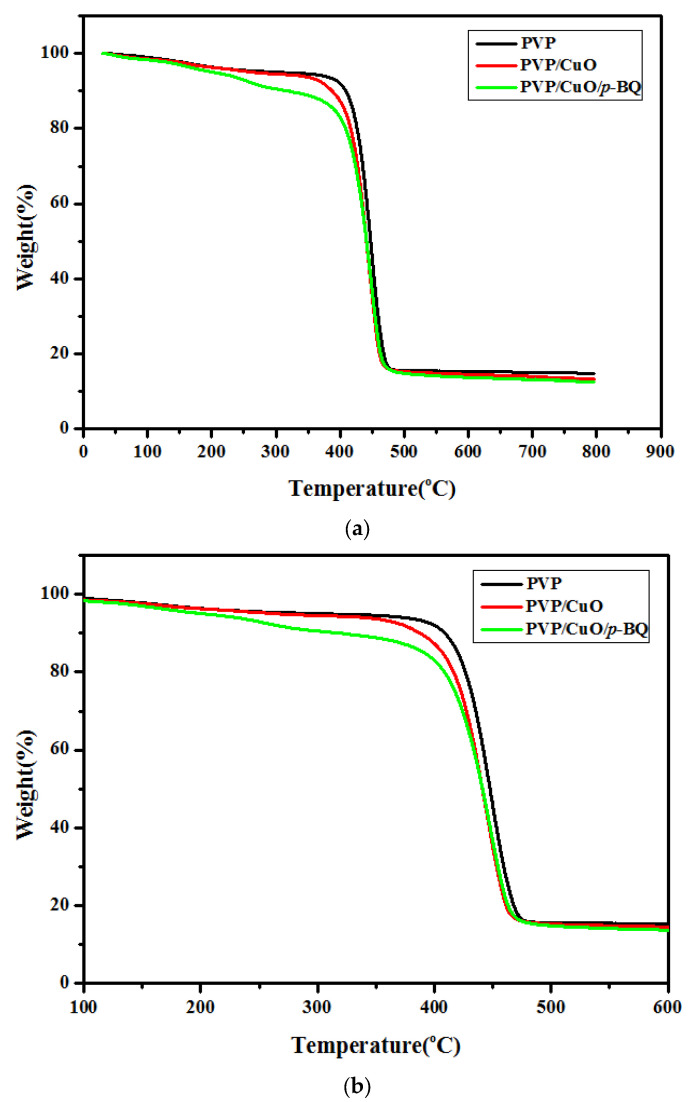
TGA curves of (**a**) PVP, PVP/CuO, and PVP/CuO/*p*-BQ composites and (**b**) enlarged graph.

The interaction between CuO and *p*-BQ and its effect on the separation performance of the PVP/CuO/*p*-BQ composite were investigated by measuring the thermal stability of PVP, PVP/CuO, and PVP/CuO/*p*-BQ. Figure 4a illustrates the temperature at which the weight reduction of neat PVP, PVP/CuO, and PVP/CuO/*p*-BQ occurred. In the case of neat PVP, weight reduction occurred at about 430 °C. On the other hand, in the case of PVP/CuO, to which CuO was added, weight reduction occurred at about 400 °C, which was a slightly lower temperature than that which caused the weight reduction of neat PVP. It can be seen that, by adding CuO, the free volume in the PVP increased, and PVP/CuO became more flexible than neat PVP. In the case of PVP/CuO/*p*-BQ, the first weight reduction occurred at about 230 °C, which was significantly lower than the weight reduction of neat PVP and PVP/CuO, and then the second weight reduction occurred at about 360 °C. This is attributable to the PVP/CuO/*p*-BQ being more flexible than the previous neat PVP and PVP/CuO. Furthermore, the free volume increased by applying *p*-BQ. The addition of *p*-BQ facilitated the dispersion of CuO. In addition, it can be seen that, since the interaction between *p*-BQ and the polymer was maintained, the free volume increased, and the chain became flexible. 

### 3.5. UV–Vis

**Figure 5:**
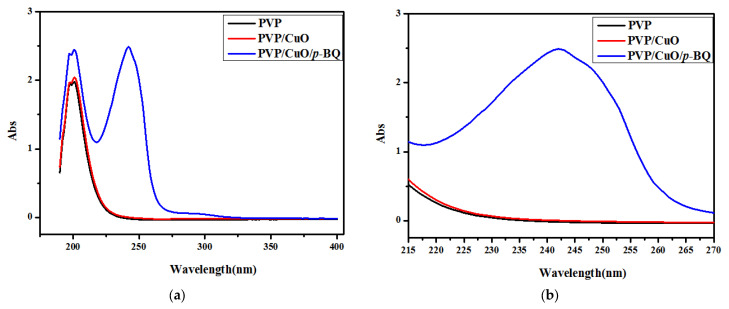
(**a**) and (**b**) UV–vis spectrum of PVP, PVP/CuO, and PVP/CuO/*p*-BQ composites.

Figure 5a,b show the number and size of copper particles for PVP and PVP/CuO/*p*-BQ measured by UV–vis spectroscopy. In the case of CuO, it is known that oscillation occurs at a wavelength of about 300 nm. In the case of PVP and PVP/CuO, oscillation was not observed at a specific wavelength, while, in the case of PVP/CuO/*p*-BQ, to which *p*-BQ was applied, oscillation was observed in a region of about 300 nm. This means that copper exists in PVP/CuO/*p*-BQ. In the PVP/CuO composite, oscillation did not occur because the particle size of copper was too large due to aggregation. Thus, in the case of PVP/CuO/*p*-BQ, it is thought that *p*-BQ plays a role in dispersing and stabilizing the CuO particles. In the case of Figure 5b, it can be observed that the graph for PVP/CuO/*p*-BQ is slightly asymmetrically distributed based on about 245 nm. Since the size of copper was not uniform, it indicates that the overall size was similar.

### 3.6. XPS (X-ray Photoelectron Spectroscopy)

**Figure 6:**
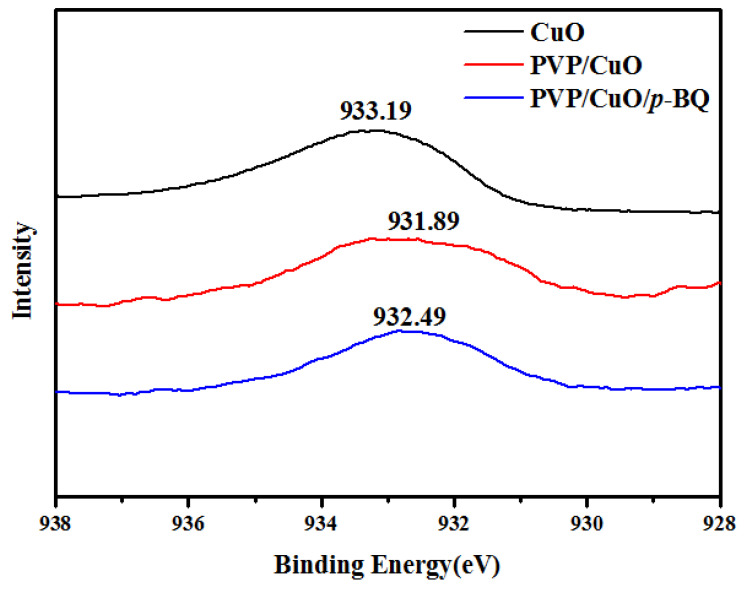
The binding energy of copper with CuO, PVP/CuO, and PVP/CuO/*p*-BQ composites.

The binding energy of copper in pure CuO and PVP/CuO and PVP/CuO/*p*-BQ was measured using X-ray photoelectron spectroscopy. The binding energy of copper in copper(Ⅱ) oxide is about 933.19 eV, as shown in Figure 6. When the copper(Ⅱ) oxide was added to PVP, the binding energy of copper decreased to about 931.89 eV. In the case of PVP/CuO/*p*-BQ, to which *p*-BQ was added, it was found that the binding energy of PVP/CuO slightly increased to 932.49 eV, higher compared to that of PVP/CuO. It can be explained by the incorporation of *p*-BQ reducing the electron density and increasing the binding energy of copper. As the surface area of copper decreased, the electron density of copper increased. In addition, it was observed that the shape was more symmetrical in the case of PVP/CuO/*p*-BQ compared to in that of PVP/CuO composite. This means that the size of the CuO stabilized by *p*-BQ was uniform, which was consistent with the UV analysis.

### 3.7. Transmission Electron Microscopy (TEM)

**Figure 7:**
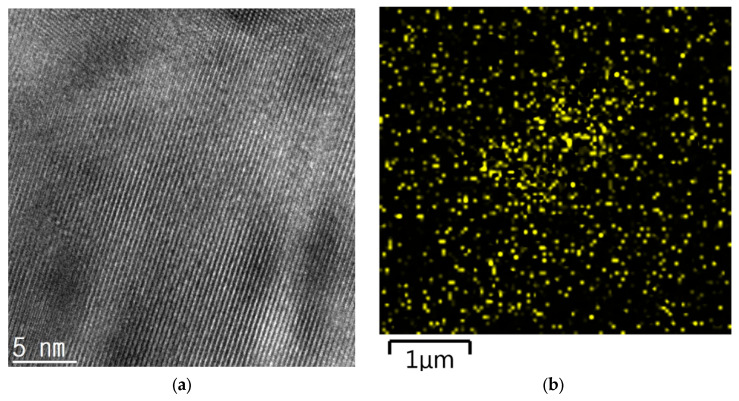
(**a**) TEM image of PVP/CuO/*p*-BQ and (**b**) EDS analysis.

The CuO particles in the PVP/CuO/*p*-BQ composite membrane were observed using TEM. In the case of Figure 7a, the surface state of CuO was observed, and Figure 7b shows the distribution of CuO particles via EDS analysis. Figure 7a,b shows that CuO particles were successfully stabilized by *p*-BQ. 

## 4. Conclusions

**Figure 8:**
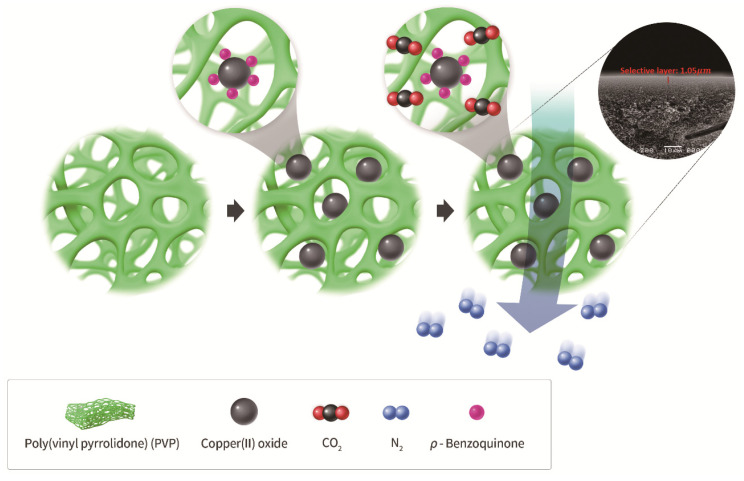
N_2_ separation by PVP/CuO/*p*-BQ composite membrane.

The composite membrane was successfully prepared by adding CuO and *p*-BQ based on a PVP polymer matrix as shown in Figure 8. The PVP/CuO/*p*-BQ composite membrane exhibited high N_2_ selectivity in the CO_2_/N_2_ separation experiment. It was measured using SEM, FT-IR, TGA, UV–vis, XPS, and TEM to find out the properties of the PVP/CuO/*p*-BQ composite membrane. From the analysis data, it was confirmed that *p*-BQ play a role in stabilizing and dispersing the size of Cu relatively uniformly compared to those of PVP/CuO. Furthermore, the electron density of Cu became reduced, and the PVP/CuO/*p*-BQ was more flexible than PVP/CuO. It can be explained through FT-IR and TGA that *p*-BQ can form an interaction between CuO and PVP. The N_2_/CO_2_ selectivity of PVP/CuO/*p*-BQ was measured using the gas separation measurement method. As a result, it can be seen that the permeance of N_2_ was 13.3 GPU, and the N_2_/CO_2_ selectivity was about 23.1 GPU. In the case of PVP/CuO, the separation performance was greatly improved due to *p*-BQ, considering that N_2_ permeance was 13.8 GPU, whereas N_2_/CO_2_ selectivity was 0.9 GPU. It may be seen that the CuO modified by *p*-BQ reduced CO_2_ transport.

## Figures and Tables

**Table 1:** Separation performance of neat PVP, PVP/CuO, and PVP/CuO/*p*-BQ composite membranes.

	N_2_/CO_2_ Selectivity	N_2_ Permeance (GPU)
Neat PVP	Not measurable	Not measurable
PVP/CuO	0.9	13.8
PVP/CuO/*p*-BQ	23.1 ± 1.8	13.3 ± 0.3

## Data Availability

Not applicable.
